# Voice and vocal fold position in men with unilateral vocal fold paralysis

**DOI:** 10.1590/S1808-86942011000600013

**Published:** 2015-10-19

**Authors:** Karine Schwarz, Carla Aparecida Cielo, Nédio Steffen, Geraldo Pereira Jotz, Jéferson Becker

**Affiliations:** 1 MSc in Human Communication Disorders - UFSM; PhD in Neurosciences - UFRGS; 2 PhD in Applied Linguistcs - PUC/RS; Adjunct Professor at the Undergraduate Program in Speech and Hearing Therapy and at the Graduate Program in Human Communication Disorders of the Federal University of Santa Maria-RS; 3 PhD - Paulista School of Medicine (UNIFESP), Professor of Otorhinolaryngology - FAMED - PUC-RS; 4 Post-Doctorate in Otorhinolaryngology - University of Pitsburgh - USA; PhD, Adjunct Professor of Otorhinolaryngology of the Medical Program of ULBRA and of the Department of Morphological Sciences of the Federal University of Rio Grande do Sul, UFRGS; 5 PhD in Medicine and Health Sciences - Catholic School of Rio Grande do Sul - PUC; Neurologist at the Lutheran University of Brazil. Federal University of Rio Grande do Sul and Catholic University of Rio Grande do Sul

**Keywords:** dysphonia, vocal cord paralysis, voice

## Abstract

**Abstract:**

The paralyzed vocal fold positioning and the degree of dysphonia are important inputs when one is deciding upon treatment options for unilateral vocal fold paralysis (UVFP).

**Objective:**

To check voice characteristics and paralyzed vocal fold position in men with UVFP.

**Materials and Methods:**

This is a retrospective historical cross-sectional cohort study, with data from 24 men with UVFP with mean age of 60.7 years, submitted to voice assessment by three speech therapists and three ENT physicians used laryngeal images to classify the position of the paralyzed vocal fold.

**Results:**

The paralyzed vocal fold was found in the paramedian position in 45.83% of the cases; in the intermediary position in 25%; lateral in 20.83%, and it was in the median position in 4.16%; the dysphonia resulting from the UVFP was characterized by moderate hoarseness, roughness and stress in the voice; breathiness (most had severe breathiness); weakness and instability(mostly mild); the position of the paralyzed vocal fold had a significant influence on the general degree of vocal deviation.

**Conclusion:**

The general degree of dysphonia is associated with the paralyzed vocal fold position; dysphonia is characterized by hoarseness, breathiness, roughness and stress of moderate to severe levels.

## INTRODUCTION

The precise incidence of vocal fold paralysis in the Brazilian Population is still unknown^1^,^2^. Notwithstanding, most vocal fold paralysis cases are among the elderly, and this is associated with a higher incidence of progressive neurological and non-laryngeal malignant diseases in this age range^2^. Vocal fold paralysis onset mean age is between 55 and 64 years^3^,^4^.

Some studies^3^,^5^ have shown that non-laryngeal malignant diseases, especially pulmonary, would be one of the main causes of vocal fold paralysis, being higher than thyroid surgery and iatrogenic causes. The second most frequent cause for such paralysis would be trauma, either surgical or not, including trauma secondary to neck surgeries, such as thyroidectomies, tracheal intubation, thoracotomy, car accidents and wounds caused by weapons^3^,^5^.

As far as gender is concerned, the literature studied does not mention the prevalence as to vocal fold mobility changes. Nonetheless, when the cause of this immobility is associated with thyroid disorder treatments, it is more frequent among females^6^.

Recent studies^7^, ^8^, ^9^ have confirmed that voice analysis, done by means of severity scales, is still a very much utilized method to assess unilateral vocal fold paralysis (UVFP) dysphonia and, despite progresses in acoustic analysis, the subjective improvement in voice noticed by the patient and the professionals involved in the treatment, is not always associated with the objective voice data.

Thus, studies^8^, ^9^, ^10^ have shown that these scales may serve as valuable tools to monitor the vocal characteristics of individuals throughout UVFP treatment or spontaneous recovery, to aid in the differentiation among the etiologies, to speculate about the paralyzed vocal fold position and, still, to differentiate the vocal characteristics between individuals with UVFP and those in the control group.

Breathiness and the difficulty to communicate effectively are the greatest losses complained by patients with UVFP^11^. Other researchers define UVFP-related dysphonia as a weak, hoarse and breathy voice, with an increase in the fundamental frequency and limitations in speech sound intensity, stiffness and with voice little projection^9,^^12^, ^13^, ^14^. Moreover, they may cause changes to swallowing, increase in the speech effort and vocal tiredness^9^,^15^,^16^.

The paralyzed vocal fold positioning and the degree of dysphonia are important factors to decide upon treatment options - surgery and speech therapy, in cases of UVFP^5,^ 15, 16, 17, 18, 19, 20. Thus, the objective of the present study was to study voice auditory-perceptive characteristics and the paralyzed vocal fold position in men with UVFP.

## METHODS

This is a historical cohort, retrospective study, carried out by studying the medical charts and voice and laryngeal tests in male patients from a private medical office, residents from Rio Grande do Sul, Brazil, diagnosed with UVFP, between January 2003 and April of 2009. This research project was previously approved by the Ethics in Research committee of the institution of origin, under protocol # 2008029.

In the present study, we included data from male patients, diagnosed with UVFP in the age range of young adults all the way to elderly, or more (according to health sciences keywords - DeCS, 2011); with a minimum paralysis time of nine months (the necessary time for nerve regeneration or a subsequent return to vocal function^16^) who underwent vocal and laryngeal assessments by the same otorhinolaryngologist.

Among the medical charts of 83 male patients, 41 were taken out because the patients had been previously submitted to speech-therapy, and we intended to check which would be the spontaneous glottic and subglottic adjustments; three had had the paralysis for less than nine months, two were not within the study's age range; two had excess secretion during the test; two had vocal fold coverage lesions: two had test which had been saved with bad image or poor sound quality; in two individuals, it was not possible to see the vocal and/or vestibular folds; one had the cricoarytenoid joint involvement as the etiology; and one had a neoplastic infiltration; one had proven neurological disease associated with the UVFP; one had vocal emission in paralytic falsetto and one was a voice professional. Thus, the study group was finally made up of 24 males with UVFP, in the age range between young adulthood and elderly patients of 80 years of age, or more, with mean age of 60.7.

As far as the patients' medical charts are concerned, we analyzed the following data: patient age and gender; past diseases, UVFP diagnosis and etiology; paralyzed side, paralysis time; tests carried out and their dates; speech therapy and professional voice usage.

Videolaryngostroboscopy was carried out with the patient seating down, and we used a *MACHIDA^®^* 70^o^, *LYC30* telescope, which was introduced in the patient's oral cavity, carried out by an ENT physician, and the images were recorded in a DVD through a GP-KS 162HD PANASONIC micro camera. During the assessment, the individual was instructed to sustain the vowel /e/ in modal vocal, after a deep inspiration, all the way to the end of expiration. In this assessment, we considered the vocal process at the point of greatest abduction and adduction as the anatomical structure of reference. The laryngeal telescope was supported on the tongue base with a mild 20 degree tilt in order to obtain the same angle in all the evaluations.

The patient's voice sampling was done by a speech therapist in the same day of the videolaryngostroboscopy, by means of uttering a stretch of spontaneous speech, reading a text and counting numbers. The vocal sample was recorded with an *MZ-R7000DPC* Sony digital sound recorder and 57 A Shure unidirectional, electro-condenser microphone in a silent room, with background noise below 50dB SPL (sound pressure level), checked by a digital sound pressure level measuring device from *RADIO SHACK^TM^.* In this assessment, the subject was standing up, with the arms extended along the body and the microphone was placed in a pedestal at 90° angle from the individual's mouth, always keeping the same distance between the microphone and the individual's mouth (ten centimeters).

The laryngeal images were edited by means of the *Vegas Movie Studio 8.0, software, from Sony* and saved in a DVD for later analysis. In order to carry out the visual-perceptive assessment of the position of the paralyzed vocal fold, we had three otorhinolaryngologists, experienced in laryngology. Each professional carried out his assessment, independently from the opinion of the others, by observing the laryngeal images during the sustained utterance of the /e/ vowel, saved in a DVD. The position of the paralyzed vocal fold was classified as follows: median, paramedian, intermediate or lateral^21^. For their assessment, the examiners were instructed to analyze the laryngeal images according to their clinical experience and they also received an instructions handbook, with an illustration of the vocal fold positions, as a model^21^. The laryngeal images of the sustained vowel /e/ were saved in DVD and randomly organized.

The auditory-perceptive evaluation was done by three speech therapists, voice experts, with much experience in this type of test. Before the beginning of the assessment, the examiners were submitted to a hearing screening, with a scanning in the frequencies between 500 and 8 KHz (kilohertz), in 25 dB (decibels), with the *VCS 2050* audiometer, from *Auditec*, in order to check for possible auditory changes which could compromise voice analysis.

After hearing screening, each speech therapist carried out her auditory-perceptive assessment, independently from the assessment of her peers, based on her clinical experience. Vocal quality was classified and quantified by means of the RASATI^22^ scale, which is an adaptation of the GRBAS American version into Portuguese, in which R relates to hoarseness; A, roughness; S, breathiness; A, weakness; T, stress and I, instability, with four levels: 0 - absence; 1 - mild; 2 - moderate and 3 - severe and noted down in a specific protocol form. In order to analyze the general level of vocal deviation, we added up the results from each item in the scale, and 18 was the highest level. The speech therapist examiners did not know about the type of disease the patients had.

For result purposes, we considered the prevailing opinion among the three examiners as far as laryngeal images and voice analyzes of the patients with UVFP are concerned. The judgment about the answers of the examiners, in both assessments, was submitted to reliability analyses, with the use for the: *Kendall* W (*Kendall's Coefficient of Concordance) approach*, which aimed at checking whether or not there was statistical significance on the agreement among the examiners.

In order to describe the sample profile, according to the study variables, we used descriptive statistics; the Binomial test was employed in order to check whether or not the etiology found in the literature also happened in the sample studied and the predominance of the paralyzed side. In comparing the categorical variables among the groups, we used the Fisher's exact test when the expected values were lower than five; in the numerical values between three or more groups we used the *Kruskal-Wallis* test. Analyzing the position of the paralyzed vocal fold, for a greater analyses consistence, we chose to group the median and paramedian positions, because of the low frequency of the median position.

All data was analyzed and computed by the *SAS System for Windows (Statistical Analysis System) software*, version 8.02. The level of significance adopted for the statistical tests was 5%.

## RESULTS

Results from the 24 patients involved in this study are depicted on tables, according to the data obtained from the charts and assessments of voice and laryngeal protocols for each patient. The data marked with an asterisk (*) was statistically significant.

Statistical tests: Binominal test to check which is the predominant etiology in the sample: *p*=0.007*;

Binominal test to check which paralyzed side prevailed in the sample: paralyzed side: *p*=0.103;

Kruskal-Wallis test: Ratio between UVFP AND GDVT: *p*=0.003*;

Kruskal-Wallis Test: Relationship between Age Range x UVFP: Fisher's Exact Test: *p*=0.430.

## DISCUSSION

As far as the etiology is concerned, the occurrence of lung cancer was significant among the male individuals with UVFP (Table 1), of the sample studied. The most common types of tumors were the epidermoid carcinomas with mediastinum metastases which, in advanced stages, involve the recurrent nerve. Patients with unilateral vocal fold paralysis, caused by lung cancer, usually have very reduced phonation time (less than three seconds) and they complain of dyspnea and great tiredness to speak. These characteristics are but an alert to refer the patient to a pneumologist. The city where this study was carried out has a reference hospital for oncology and lung cancer treatment and some patients we referred from this institu-tion. Nonetheless, it is believed that this data can be also associated to the fact that lung cancer is the number one cause of death by cancer among men from Rio Grande do Sul, and such state has the highest incidence and mortality rates in the country^23^. Other studies have also revealed that the non-laryngeal malignant disease, especially the pulmonary one, would be one of the main causes of vocal fold paralysis, being higher that thyroid surgery and iatrogenic causes^3^,^5^. In the authors' clinical practice, the phenomenon can also be informally observed, which converge with the significant results seen in the present study.

**Table 1:** Patient characterization as to UVFP etiology, paralyzed side, auditory-perceptive evaluation with the RASATI scale, general degree of vocal deviation and position of the paralyzed vocal fold.

PATIENT	AGE RANGE	ETIOLOGY*	PARALYZED SIDE	RASATI	GGDV*	UVFP*
1	ELDERLY	CHEST SURGERY	LEFT	R3A2S3A2T0I1	11	PARAMEDIAN
2	ELDERLY	LUNG CANCER*	LEFT	R3A2S2A1T1I1	10	PARAMEDIAN
3	PRE-ELDERLY	LUNG CANCER	LEFT	R2A3S3A2T2I1	13	INTERMEDIARY
4	ELDERLY	AORTIC ANEURISM	LEFT	R1A3S3A3T2I1	13	LATERAL
5	PRE-ELDERLY	IDIOPATHIC	RIGHT	R2A3S3A2T2I1	13	PARAMEDIAN
6	ADULT	IDIOPATHIC	LEFT	R2A2S2A1T0I1	8	PARAMEDIAN
7	ELDERLY	LUNG CANCER	LEFT	R2A2S3A3T1I0	11	INTERMEDIARY
8	PRE-ELDERLY	VAGUS NERVE SCHWANNOMA	RIGHT	R1A2S3A3T2I0	11	PARAMEDIAN
9	ADULT	CHEST SURGERY	LEFT	R2A3S3A1T2I0	11	PARAMEDIAN
10	ADULT	CAROTID ANEURISM	RIGHT	R3A2S3A2T2I1	13	LATERAL
11	ADULT	IDIOPATHIC	RIGHT	R2A2S3A1T1I0	9	PARAMEDIAN
12	PRE-ELDERLY	LUNG CANCER	LEFT	R3A3S3A3T2I2	16	LATERAL
13	ELDERLY	THYROID SURGERY	LEFT	R2A3S3A3T2I1	14	LATERAL
14	ELDERLY OF 80 OR +	THYROID SURGERY	LEFT	R3A3S2A2T2I1	13	INTERMEDIARY
15	ELDERLY	SKULL-BASE SURGERY	RIGHT	R2A2S1A1T1I1	8	INTERMEDIARY
16	ADULT	POST DISK HERNIATION SURGERY	RIGHT	R1A1S1A0T0I0	3	PARAMEDIAN
17	ELDERLY	IDIOPATHIC	LEFT	R2A2S1A0T1I2	8	PARAMEDIAN
18	ELDERLY	THYROID SURGERY	RIGHT	R2A2S3A2T2I1	12	MEDIAN
19	ELDERLY	CAROTID SURGERY	LEFT	R2A3S3A2T2I1	13	INTERMEDIARY
20	PRE-ELDERLY	AORTIC ANEURISM	LEFT	R2A2S3A1T2I2	12	PARAMEDIAN
21	PRE-ELDERLY	FIREARM	LEFT	R2A3S3A3T3I2	16	LATERAL
22	PRE-ELDERLY	LUNG CANCER	LEFT	R2A2S2A1T2I1	10	PARAMEDIAN
23	ELDERLY	CAROTID SURGERY	LEFT	R3A3S3A0T0I2	11	INTERMEDIARY
24	PRE-ELDERLY	LUNG CANCER	RIGHT	R2A1S1A1T1I1	7	MEDIAN

RASATI, where R: hoarseness; A: roughness; S: breathiness; A: weakness; T: stress and I: instability.

RASATI scale levels: 0: absence; 1: mild; 2: moderate and 3: severe.

GGDV: general degree of local deviation (0-18).

PPVP: paralyzed vocal fold position.

Age range: ADULT (19-44 YEARS OF AGE); PRE-ELDERLY (45-64 YEARS OF AGE); ELDERLY(65-79 YEARS OF AGE); ELDERLY OF 80 OR + (80 YEARS OF AGE OR MORE) Source: Health sciences keywords 2011 (DeCS, 2011).

In the present investigation, the left side paralysis was frequent, and there was no significant relationship between the paralyzed side and the other variables in the study (Table 1). Some of the UVFP studies are in agreement with such finding^24^,^25^. The greatest occurrence of lesions on the left side is associated with the difference in the anatomical course of the recurrent laryngeal nerve, which is long and partially intrathoracic, and it can also be affected by mediastinal diseases. When the paralysis predominates in the right side, usually the UVFP etiology is associated to a thyroidectomy sequela^14^.

As far as the age range is concerned, we noticed a predominance of the elderly population, with a mean of 60.7 years (Table 1). According to the literature studied, the mean age of paralysis onset is between 55 and 64 years^2^,^5^. Thus, it is among the elderly population that we find the greatest prevalence of vocal fold paralyses, and it is associated with the greater incidence of progressive neurologic disorders and non-laryngeal malignant diseases in this group. As we correlate the age range with the other variables in the present study: PPVP, GGDV, RA-SATI, paralyzed side and etiology did not show statistical significance.

Among dysphonia evaluation measures, vocal quality analyses is very much used, having seen that voice is an acoustic phenomenon and its auditory perception, even when influenced by subjective characteristics of the listener, is the most proper tool for its assessment, since it identifies the change characteristic and severity. Thus, it is recommended to use scales for an auditory-perceptive voice test, since it enables for a common and consistent language, which makes possible to compare studies. The GRBAS scale is considered by many authors as the most utilized^22^,^26^. In one study^27^, it was noticed that in the subjective analysis of the voice, there were no significant differences among the assessments made by the experienced and inexperienced examiners and that the degree of dysphonia is one of the most reliable indicators. In the present study, the professionals who did the voice auditory-perceptive analysis were experienced voice experts, and the aspects which had the greatest reliability were hoarseness, breathiness, weakness and stress (Table 2).

**Table 2:** Agreement analysis between the auditory-perceptive assessments of the voices by the speech therapist examiners by means of the RASATI scale.

RASATI	W	P
Hoarseness	0.519	*p*=0.043*
Roughness	0.438	*p*=0.418
Breathiness	0.824	*p*<0.001*
Weakness	0.866	*p*<0.001*
Stress	0.537	*p*=0.032*
Instability	0.469	*p*=0.481

Legend: W=Kendall's agreement coefficient

Dysphonia stemming from the UVFP is usually characterized by weak, hoarse and breathy voice, with an increase in the fundamental frequency and limitations to the speech sound intensity^6^,^12^,^13^. In the present study, we noticed a predominance of hoarseness, roughness and stress (higher frequency of the moderate degree); breathiness (higher frequency of the moderate degree), weakness and instability (higher frequency of the mild degree) and higher vocal deviation general degree (Table 1). In one study^12^, the examiners considered that the 40 patients with UVFP had moderate to severe dysphonia, being mainly characterized by the values of breathiness (B), followed by the values of roughness (R), according to the GRBASI scale. The values regarding weakness (A) were higher than those for stress (S), which the authors associated to the fact that a possible glottic failure may cause the impression of weakness, even when stress is present. In the present study, weakness was considered severe in six individuals, and stress was severe in one individual. Weakness may be associated with the age range (most were elderly) and to the prevalent UVFP etiology among the patients (lung cancer), variables which could impact on respiratory control and the paralyzed vocal fold tonus, and cause a weaker voice. Studies^6^,^12^,^13^ which assessed the general degree of vocal deviation in UVFP patients, by means of auditory-perceptive vocal scales have also found high indices. One study^28^ in which 28 UVFP patients were assessed together with a group of 12 patients without vocal complaints, the authors noticed that the degree of change (G), breathiness (B) and weakness (A) were significantly correlated with objective measures, obtained by means of the *Evaluation Vocale Assistée* software, which indicated high *Jitter Shimmer* rates.

Pioneer studies about the vocal fold have indicated that the degree of symptom severity and the lesion site could be confirmed based on vocal fold position^24^,^29^. More up-to-date studies have shown that it is not possible to diagnose the lesion site based solely on vocal fold position, and that these factors are not highly correlatable^30^,^31^. In our study, the paramedian and intermediate positions were the ones most commonly found (Table 1), in agreement with the results from other studies^2^,^24^. Other authors^25^,^32^ found results which differ from the ones we found, since they argued that the median and paramedian positions were the most frequently found in their sample. The mechanisms responsible for the vocal fold positions are still unknown; notwithstanding, there are studies arguing that it may be affected by the degree of reinervation, as well as by the fibrosis of the denervated muscles^31^,^33^.

One study showed that UVFP signs and symptoms may vary depending on the position of the paralyzed vocal fold, with higher or lower degrees of breathiness, hoarseness and diplophonia^12^. However, based on clinical experience, other authors^20^ believe that it is not always that the paralyzed vocal fold position corresponds to the degree of dysphonia and that the degree of dysphonia matches the degree of vocal fold atrophy and laxity, as well the overlapping of the healthy vocal fold and the laryngeal constriction. In the present study, as we related the paralyzed vocal fold position to the general degree of deviation, we noticed that dysphonia was significantly higher in the lateral and intermediary positions (Table 1). Thus, we believe that the higher the glottal failure caused by the non-median positions, the greater will be the patient's vocal limitations.

The assessment of a paralyzed vocal fold is no easy task for the ENT physician (Table 3). Even the most experienced may have difficulties in assessing the characteristics of a patient with unilateral vocal fold paralysis. In one study^34^, the aspects with had the most reliability among the three ENT examiners in the pre and post-medialization assessment of the paralyzed vocal fold were: the paralyzed vocal fold position, its closeness to the vestibular fold (present or absent) and the type of vestibular fold approximation (contralateral, ipsilateral or absent). The evaluation of the type of glottic closure, vocal fold free margin, overlapping of the healthy vocal fold to beyond the mid line and the type of constriction of the laryngeal vestibule did not have reliability among the examiners in the two moments.

**Table 3:** Agreement analysis between the evaluations of the laryngeal images by the ENT examiners.

Laryngeal image	W	P
PPVP	0.813	*p*<0.001*

PPVP=paralyzed vocal fold position; W=Kendal's agreement coefficient

In another study^28^, one of the few found in the literature involving a double blind assessment of laryngeal images by ENT physicians, three examiners, very experienced in the analysis and interpretation of laryngeal images carried out the assessment of patients with unilateral vocal fold paralysis in order to study supraglottic configuration. Each examiner studied the recorded video twice, at different times and, in order to enable an estimate of reliability among the examiners, the data was compared and considered correct when at least three examiners agreed upon the results. Thus, we believe we need more studies in order to standardize laryngeal image assessment protocols based on visual-perceptive analysis.

## CONCLUSION

In the sample studied we observed that:

• In UVFP, the general degree of dysphonia is influenced by the paralyzed vocal fold position, being significantly higher in the lateral and intermediary positions;
• Dysphonia caused by UVFP is mainly characterized by moderate to severe hoarseness, breathiness, roughness and stress.

## References

[1] Melo ECM, Brito LL, Brasil OCO, Behlau M, Melo DM. (2001). Incidência de lesões laríngeas não neoplásicas em pacientes com queixas vocais.. Rev Bras Otorrinolaringol..

[2] Ahmad S, Muzamil A, Lateef M. (2002). A Study of incidence and etiopathology of vocal cord paralysis.. Indian J Otolaryngol Head Neck Surg..

[3] Terris D, Arnstein D, Nguyen H. (1992). Contemporary evaluation of unilateral vocal cord paralysis.. Otolaryngol Head Neck Surg..

[4] Herrington-hal BL, Stemple JC, Niemi KR, Mchone MM. (1988). Description of laryngeal pathologies by age, sex and occupation in a treatment-seeking sample.. J Speech Hear Disord..

[5] Benninger MS, Crumley RL, Ford CN, Gould WJ, Hanson DG, Ossoff RH (1994). Evaluation and treatment of the unilateral paralyzed vocal fold.. Otolaryngol Head Neck Surg..

[6] Mangilli LD, Amoroso MRM, Nishimoto IN, Barros APB, Carrara-de-Angelis E. (2008). Voz, deglutição e qualidade de vida de pacientes com alteração de mobilidade de prega vocal unilateral pré e pós-fonoterapia.. Rev Soc Bras Fonoaudiol..

[7] Franco RA, Andrus JG. (2009). Aerodynamic and acoustic characteristics of voice before and after adduction arytenopexy and medialization laryngoplasty with gore-tex in patients with unilateral vocal fold immobility.. J Voice..

[8] Little MA, Costello DAE, Harries ML. (2011). Objective dysphonia quantification in vocal fold paralysis: comparing nonlinear with classical measures.. J Voice..

[9] Nemr K, Amar A, Abrahão M, Leite GCA, Köhle J, Santos AO (2005). Comparative analysis of perceptual evaluation, acoustic analysis and indirect laryngoscopy for vocal assessment of a population with vocal complaint.. Braz J Otorhinolaryngol..

[10] Leydon C, Bielamowicz S, Stager SV. (2005). Perceptual ratings of vocal characteristics and voicing features in untreated patients with unilateral vocal fold paralysis.. J Commun Disord..

[11] Alves CB, Loughran S, MacGregor FB, Dey JI, Bowie LJ. (2002). Bioplastique medialization therapy improves the quality of life in terminally ill patients with vocal cord palsy.. Clin Otolaryngol Allied Sci..

[12] Schindler A, Bottero A, Capaccio P, Ginocchio D, Adorni F, Ottaviani F. (2008). Vocal improvement after voice therapy in unilateral vocal fold paralysis.. J Voice..

[13] Smith ME, Berke GS, Gerratt BR, Kreiman J. (1992). Laryngeal paralysis: theoretical considerations and effects on laryngeal vibration.. J Speech Hear Res..

[14] Patel R, Parsram KS. (2005). Acoustic analysis of subjects with vocal cord paralysis.. Indian J Otolaryngol Head Neck Surg..

[15] Selber J, Sataloff R, Spiegel J, Heman-Ackah Y. (2003). Gore-Tex medialization thyroplasty: objective and subjective evaluation.. J Voice..

[16] Boone DR, Mcfarlane SC. (1994). Voz e a terapia vocal..

[17] Kimura M, Nito T, Sakakibara KI, Tayama N, Niimi S. (2008). Clinical experience with collagen injection of the vocal fold: A study of 155 patients.. Auris Nasus Larynx..

[18] Bergamini G, Alicandri-Ciufelli M, Molteni G, Villari D, Luppi M, Genovese E (2010). Therapy of unilateral vocal fold paralysiswith polydimethylsiloxane injection laryngoplasty: our experience.. J Voice..

[19] Milstein CF, Akst LM, Hicks MD, Abelson TI, Strome E. (2005). Long-term effects of micronized Alloderm injection for unilateral vocal fold paralysis.. Laryngoscope..

[20] Brent E. Richardson, Robert W. Bastian. (2004). Clinical evaluation of vocal fold paralysis.. Otolaryngol Clin North Am..

[21] Greene CLM. (1983). Distúrbios da voz..

[22] Pinho SMR, Pontes P. (2008). Músculos Intrínsecos da Laringe e Dinâmica Vocal..

[23] Castro MSM, Vieira VA, Assunção RM. (2004). Padrões espaço-temporais da mortalidade por câncer de pulmão no Sul do Brasil.. Rev Bras Epidemiol..

[24] Dedo HH. (1970). The paralyzed larynx: an electromyographic study in dogs and humans.. Laryngoscope..

[25] Pinho SMR, Pontes PAL, Gadelha ME, Biasi N. (1999). Vestibular vocal behavior during phonation in unilateral vocal fold paralysis.. J Voice..

[26] Behrman A. (2004). Evidence-based treatment of paralytic dysphonia: making sense of outcomes and efficacy data.. Otolaryngol Clin North Am..

[27] De Bodt MS, Wuyts FL, de Van Heyning PH, Croux C. (1997). Testrests study if the GRBAS scales: Influence of experience and professional background on perceptual rating of voice quality.. J Voice..

[28] Morsomme D, Jamart J, Wery C, Giovanni A, Remacle M. (2001). Comparison between the GIRBAS Scale and the acoustic and aerodynamic measures provided by EVA for the assessment of dysphonia following unilateral vocal fold paralysis.. Folia Phoniatr Logop..

[29] Meurman OH. (1950). Theories of vocal cord paralysis.. Acta Otolaryngol..

[30] Woodson GE. (1993). Configuration of the glottis in laryngeal paralysis. I: clinical study.. Laryngoscope..

[31] Koufman JA, Walker FO, Joharji GM. (1995). The cricothyroid muscle does not influence vocal fold position in laryngeal paralysis.. Laryngoscope..

[32] Bortoncelo S, Behlau M, Pontes P., Behlau M., e Gasparine G (2006).

[33] Crumley RL. (1994). Unilateral recorrent laryngeal nerve paralysis.. J Voice..

[34] Schwarz K, Cielo CA, Steffen N, Becker J, Jotz GP. (2011). Voice and laryngeal configuration of men with unilateral vocal fold paralysis before and after medialization.. J Voice..

